# Lenalidomide or bortezomib as maintenance treatment remedy the inferior impact of high-risk cytogenetic abnormalities in non-transplant patients with newly diagnosed multiple myeloma: a real-world multi-centered study in China

**DOI:** 10.3389/fonc.2023.1028571

**Published:** 2023-04-20

**Authors:** Zhe Zhuang, Ying Tian, Lei Shi, Dongmei Zou, Ru Feng, Wei-wei Tian, Hong Yu, Fei Dong, Aijun Liao, Yanping Ma, Qinhua Liu, Shuangjiao Liu, Hongmei Jing, Rong Fu, Liang-ming Ma, Hui Liu, Wanling Sun, Li Bao, Yin Wu, Wenming Chen, Junling Zhuang

**Affiliations:** ^1^ Department of Hematology, Peking Union Medical College Hospital, Chinese Academy of Medical Sciences, Beijing, China; ^2^ Department of Hematology, Beijing Chao-Yang Hospital Capital Medical University, Beijing, China; ^3^ Department of Hematology, Beijing University, Beijing Jishuitan Hospital, Beijing, China; ^4^ Department of Hematology, Xuanwu Hospital Capital Medical University, Beijing, China; ^5^ Department of Hematology, Beijing Hospital, National Center of Gerontology, Institute of Geriatric Medicine, Chinese Academy of Medical Science, Beijing, China; ^6^ Department of Hematology, Shanxi Bethune Hospital of Shanxi Medical University, Taiyuan, China; ^7^ Department of Hematology, Tianjin Medical University General Hospital, Tianjin, China; ^8^ Department of Hematology, Peking University Third Hospital, Beijing, China; ^9^ Department of Hematology, Shengjing Hospital of China Medical University, Shenyang, China; ^10^ Department of Hematology, The Second Hospital of Shanxi Medical University, Taiyuan, China; ^11^ Department of Hematology, First Affiliated Hospital of Anhui Medical University, Hefei, China

**Keywords:** maintenance therapy, high-risk disease, real-world study, multicenter, multiple myeloma

## Abstract

Maintenance treatment is a pivotal part in the whole process management of multiple myeloma (MM), which further deepens response and improves survival. However, evidence of maintenance in non-transplant MM patients is inadequate in real-world practice. Here, we retrospectively analyzed the efficacy and survival of 375 non-transplant MM patients from 11 centers between 2010 and 2021 in north China. After a median of seven cycles of front-line regimens, there were 141, 79, and 155 patients receiving lenalidomide maintenance (L-MT), bortezomib maintenance (B-MT), or thalidomide maintenance (T-MT), respectively. Patients on L-MT and B-MT had significantly greater proportions of high-risk cytogenetic abnormalities (HRCAs) detected by fluorescence *in situ* hybridization (FISH), which was defined as 1q21 gain, 17p deletion, adverse immunoglobulin heavy chain (IgH) translocations. Although the progression-free survival (PFS) and overall survival (OS) were comparable among the three groups, L-MT and B-MT remedied the negative impact of HRCAs on survival (PFS of patients with HRCAs vs. patients without HRCAs: L-MT, 26.9 vs. 39.2 months, p=0.19; B-MT, 20.0 vs. 29.7 months, p=0.36; OS not reached in all groups). Patients with HRCAs in the T-MT group presented inferior clinical outcomes compared to standard-risk patients (PFS, 12.1 vs. 22.8 months, p=0.02, HR=1.8, 95% CI 1.0–3.4; OS, 54.9 months vs. NR, p<0.001, HR=3.2, 95% CI 1.5–7.0). Achieving complete response (CR) after induction therapy led to superior PFS compared to other degrees of response, regardless of maintenance medication. Furthermore, maintenance duration over 24 months correlated with favorable survival. Due to the large gap of transplant eligibility in China, optimizing maintenance therapy is important for non-transplant MM patients. In this real-world multi-centered study, our findings suggest that clinicians prefer to prescribe lenalidomide or bortezomib as maintenance therapy in high-risk settings, which are superior to thalidomide in non-transplant MM patients. Achievement of CR and maintenance duration over 2 years are positive factors that influence survival.

## 1 Introduction

Multiple myeloma (MM) is the second most prevalent hematological malignancy (1, 2), with an estimated incidence of 0.88–1.17/100,000/year in China (3), and remains incurable despite treatment advances. The incidence of MM has increased, mainly related to aging and the availability of diagnostic approaches. Substantial developments have largely contributed to the improving clinical outcomes of MM patients in the last two decades, such as emerging novel agents, eligibility of autologous stem cell transplantation (ASCT), and strategy of maintenance therapy (4). Even in the era of novel multi-drug induction, maintenance therapy is still one of the essential parts in the whole process management, which sustains and upgrades the response depth and prolongs survival, especially progression-free survival (PFS) (5). Various randomized controlled trials (RCTs) have demonstrated the efficiency and safety of maintenance with a strong immunomodulatory drug (IMiD), lenalidomide; some suggested the benefits of maintenance with bortezomib in patients with high-risk cytogenetics like deletion 17p (6–11). Thalidomide as maintenance has not been recommended in some authoritative guidelines because of the alternative IMiD lenalidomide or its limits of improving PFS and overall survival (OS) in high-risk population (12, 13).

Due to inadequate access to melphalan, ASCT rate is relatively low in Chinese transplant eligible newly diagnosed multiple myeloma (NDMM) patients. Therefore, maintenance after front-line induction seems crucial. However, there has been no adequate data presenting the current situation of maintenance therapy in China, especially in non-transplant patients (14, 15). As economic aspects are considered, thalidomide is still administrated in China. Therefore, we conducted this multi-centered retrospective study on the efficacy and safety of lenalidomide, thalidomide, and bortezomib as maintenance therapy, aiming to delineate the present status of maintenance strategies in real practice in China.

## 2 Method

### 2.1 Patients and study design

Medical records were extracted to build the Northern China MM Registry database, including those from 11 tertiary hospitals. From the database, non-transplant MM patients diagnosed between 1/1/2010 to 31/7/2021 were screened. Demographic information, international staging system (ISS), revised-ISS (R-ISS), lactic dehydrogenase (LDH), front-line regimens, treatment responses, and adverse events were recorded. CD138-positive marrow cells were sorted for fluorescence *in situ* hybridization (FISH). Cytogenetic abnormalities (CA) were detected, including amplification of 1q21 (1q21+), deletion 17p (17p−), t(4,14), t(14,16), and t(11,14). High-risk CAs (HRCAs) were defined as 1q21+, 17p−, t(4,14), and t(14,16). Those receiving maintenance therapy by lenalidomide or bortezomib or thalidomide (with or without dexamethasone) after front-line induction therapy and achieving partial response (PR) or better were retrospectively enrolled. The timing and doses of maintenance regimens depended on the respective practice routines. Typically, thalidomide was administrated at 75–150 mg/day. The dose of lenalidomide was 25 mg every other day or 10 mg daily (according to the available dosages, renal function, or adverse reactions), on day 1–21 of 28-day cycle. Bortezomib (1.3 mg/m^2^ s.c.) was administered every 2 weeks or four doses every 3 months, mainly due to the inconvenience of subcutaneous injection of bortezomib. Dexamethasone was given along in some patients. This study was approved by the Institutional Ethics Committee of Peking Union Medical College Hospital and the Institutional Ethics Committee of the participating centers of the Northern China MM Registry.

### 2.2 Response evaluation

The International Myeloma Working Group 2016 efficacy criteria was used to evaluate the response of maintenance therapy (16). The treatment response was evaluated at each follow-up, including stringent complete response (sCR), complete response (CR), very good partial response (VGPR), partial response (PR), minimal response (MR), stable disease (SD), and progression disease (PD).

### 2.3 Safety evaluation

Electronic medical records were reviewed, and adverse events during maintenance therapy were recorded and graded according to National Cancer Institute’s Common Terminology Criteria for Adverse Events (version 4.03, 2010).

### 2.4 Statistical analysis

SPSS Statistics software (version 20.0; SPSS Inc., Chicago, IL, USA) was used to conduct all statistical analyses. We used the chi-squared test to compare the frequency distributions of categorical variables and one-way ANOVA to compare the numerical variables. Progression-free survival (PFS) time was measured from the initiation of maintenance regimens to the date of disease progression (PD), death, maintenance discontinuation due to toxicity, or the last follow-up. Overall survival (OS) was calculated from the initiation of maintenance regimens to the time of death or the last follow-up. The results reported were as of May 2022. Median PFS and median OS were assessed with the Kaplan–Meier method and compared with the log-rank test. Possible prognostic factors were first screened with univariate analysis; then, a multivariate analysis was conducted with the Cox proportional hazard regression model to ascertain independent prognostic factors. *p* < 0.05 was considered of statistical significance.

## 3 Results

### 3.1 Patient demographics

A total of 375 non-transplant patients achieving PR or better after front-line induction were finally recruited in the study, including 141 with lenalidomide (L-MT), 79 with bortezomib (B-MT), and 155 with thalidomide (T-MT). Their baseline characteristics at diagnosis are reported in **Table 1**. The gender ratio, age, paraprotein type, ISS, and R-ISS were comparable (**Table 1**). As a distinct bias, the proportion of HRCAs at diagnosis was remarkably lower in the thalidomide group (n=33, 34.0%; p<0.01), compared with 60 (58.8%) patients in lenalidomide-MT and 41 (62.1%) in bortezomib-MT. There were more “double-hit” patients containing any two HRCAs in the bortezomib group (21.2%, p<0.01) than those in the lenalidomide (7.4%) or thalidomide (2.1%) groups. To be noticed, the FISH analyses were performed in each participating center with similar techniques using CD138+ magnetic beads.

**Table 1:** Baseline demographic characteristics in non-transplant NDMM patients with lenalidomide, bortezomib, or thalidomide maintenance.

		Lenalidomide N= 141	Bortezomib N= 79	Thalidomide N= 155	*p*-value
**Gender (male%)**		76 (53.9%)	40 (50.6%)	91 (58.7%)	0.46
**Age (median ± SD/year)**		63 ± 9.5	64 ± 10.6	62 ± 10.2	0.34
**Paraprotein isotype (%)**	IgG	60 (42.6%)	26 (32.9%)	72 (46.5%)	0.25
	IgA	29 (20.6%)	17 (21.5%)	33 (21.3%)	
	IgD	6 (4.2%)	4 (5.1%)	5 (3.2%)	
	Light Chain	37 (26.2%)	29 (36.7%)	40(25.8%)	
	Other or NA	9 (6.4%)	3 (3.8%)	5 (3.2%)	
**ISS**	I	27 (19.1%)	16 (20.3%)	36 (23.2%)	0.65
	II	43 (30.5%)	24(30.4%)	37 (23.9%)	
	III	62 (44.0%)	30 (38.0%)	67 (43.2%)	
	NA	9 (6.4%)	9 (11.4%)	15 (9.7%)	
**R-ISS**	I	16/108 (14.8%)	7/66 (10.6%)	14/122 (11.5%)	*0.02
	II	73/108 (67.6%)	42/66 (63.6%)	98/122 (80.3%)	
	III	19/108 (17.6%)	17/66 (25.8%)	10/122 (8.2%)	
**HRCAs**		60/102 (58.8%)	41/66 (62.1%)	33/97 (34.0%)	*<0.01
**Cytogenetic abnormalities**	Gain(1q21)	47/104 (45.2%)	30/68 (44.1%)	28/110 (25.5%)	*<0.01
	Del(17p)	16/94 (17.0%)	11/66 (16.7%)	6/108 (5.6%)	*0.02
	t(4,14)	10/91 (11.0%)	12/64 (18.8%)	1/92 (1.1%)	*<0.01
	t(14,16)	7/90 (7.8%)	3/64 (6.5%)	0/92 (0%)	*0.03
	Double hit	7/95 (7.4%)	14/66 (21.2%)	2/97 (2.1%)	*<0.01
**Response prior to MT**	sCR+CR	61 (43.3%)	36 (45.6%)	61 (39.4%)	*<0.01
	VGPR	41 (29.0%)	16 (20.2%)	66 (42.6%)	
	PR	39 (27.7%)	27 (34.2%)	28 (18.0%)	

CR, complete response; HRCAs, high-risk cytogenetic abnormalities; ISS, International Staging System; MT, maintenance therapy; PR, partial response; R-ISS, revised-ISS; sCR, stringent complete response; VGPR, very good partial response.

*p < 0.05.

The front-line therapies are listed in **Table 2**, which were not comparable in the three groups. A total of 125 (88.6%) patients received bortezomib-containing regimens prior to lenalidomide maintenance, 75 (97.4%) prior to bortezomib maintenance, while 96 (61.9%) prior to thalidomide-MT. Thirty-seven (26.2%) patients received lenalidomide-containing regimens prior to lenalidomide-MT, 10 (12.7%) prior to bortezomib-MT, while only 2 (1.3%) prior to thalidomide-MT. The median cycles of induction regimens were seven before maintenance. In the patients receiving lenalidomide-MT, 102 (72.3%) achieved VGPR or better (≥VGPR) response before the initiation of maintenance, with 61 (43.3%) patients achieving CR or sCR. In the bortezomib-MT group, 52 (67.8%) patients achieved ≥VGPR and 36 (45.6%) with CR or sCR. The numbers were 127 (82.0%) and 61 (39.4%) in the thalidomide-MT group, respectively.

**Table 2:** Front-line regimens in patients with different maintenance treatment.

Front-line regimens	Thalidomide N= 155	Lenalidomide N= 141	Bortezomib N= 79	*p*-value
**VRD**	0	27 (19.1%)	8 (10.1%)	*<0.01
**B-based (without IMiDs)**	96 (61.9%)	98 (69.5%)	69 (87.3%)	
**L-based (without PIs)**	2 (1.3%)	10 (7.1%)	2 (2.6%)	
**Others**	57 (36.8%)	6 (4.3%)	0	

B, bortezomib; L, lenalidomide; PIs, proteasome inhibitors; VRD, bortezomib, lenalidomide, and dexamethasone. * p < 0.05.

### 3.2 Outcomes of patients on lenalidomide, bortezomib, or thalidomide maintenance

**Figure 1:**
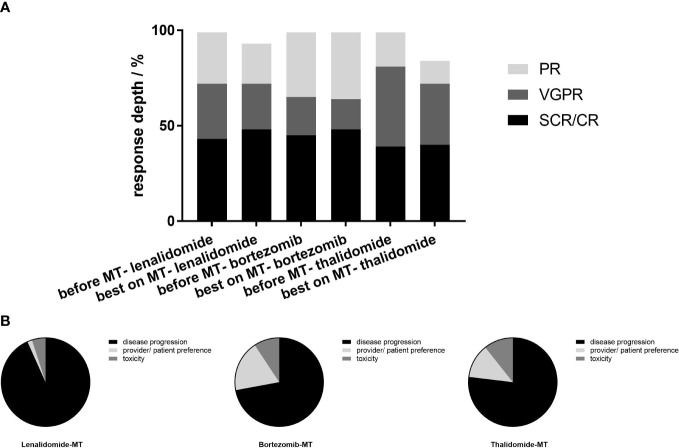
Best response before and during maintenance and reasons for discontinuing maintenance. **(A)** In the 141 patients on lenalidomide-MT, 61 (43.3%) achieved CR or sCR prior to the initiation of maintenance, 41 (29%) achieved VGPR, while 39(27.7%) achieved PR. In the bortezomib-MT group (n=79), 36(45.6%) achieved CR or sCR prior to the initiation of maintenance, 16 (20.3%) achieved VGPR, and 27 (34.2%) achieved PR. In the thalidomide-MT group (n=155), the ratio was 39.4% (n=61), 42.6% (n=66), and 18.0% (n=28), retrospectively. Best response on maintenance is shown in the adjacent column. On lenalidomide maintenance, 48.2% achieved CR or sCR, 24.8% achieved VGPR, and 21.3% achieved PR. In the bortezomib maintenance group, 48.1%, 16.5%, and 35.4% achieved CR or sCR, VGPR, and PR, respectively, and 40.6%, 32.3%, and 12.3% in patients on thalidomide maintenance. **(B)** The main reasons for discontinuing maintenance were progression (93.3%, 72.1%, and 76.9% for lenalidomide-MT, bortezomib-MT, and thalidomide-MT, respectively), provider/patient preference (1.7%, 18.6%, and 12.4%), and toxicity (5.0%, 9.3%, and 10.7%).

The median follow-up durations since maintenance were 24.0, 24.8, and 42.5 months on lenalidomide-MT, bortezomib-MT, and thalidomide-MT, respectively. Further deepening of response (improvement in IMWG response category) was recorded in 15 (10.6%) patients on lenalidomide-MT, 6 (7.6%) patients on bortezomib-MT, and 7 (4.5%) patients on thalidomide-MT (**Figure 1A**). At last follow-up, 60, 43, and 121 patients had discontinued maintenance therapy. The main reasons for discontinuing maintenance therapy were disease progression (93.3%, 72.1%, and 76.9%), provider/patient preference (1.7%, 18.6%, and 12.4%), and unacceptable toxicity despite dose modification (5.0%, 9.3%, and 10.7%) (**Figure 1B**).

**Figure 2:**
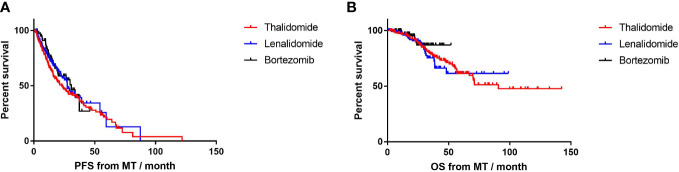
Progression-free survival (PFS) and overall survival (OS) in patients receiving lenalidomide, bortezomib, and thalidomide as maintenance. **(A)** The median PFS of lenalidomide, bortezomib, and thalidomide maintenance was 27.4, 30.8, and 23.2 months, respectively. **(B)** The median OS of lenalidomide, bortezomib, and thalidomide maintenance was not reached, not reached, and 90.7months, respectively.

Until the end of the follow-up, 60 (42.6%) patients on lenalidomide-MT, 31 (39.2%) patients on bortezomib-MT, and 102 (65.8%) patients on thalidomide-MT experienced their first relapse (**Figure 2A**). The median PFS from maintenance was 27.4, 30.8, and 23.2 months, respectively (p=0.38). The median duration of maintenance treatment was 16.0, 15.6, and 16.0 months, respectively. A total of 23 (16.3%) patients with lenalidomide-MT, 7 (8.9%) patients on bortezomib-MT, and 51 (32.9%) patients with thalidomide-MT died (**Figure 2B**). The median OS from maintenance was not reached in lenalidomide-MT or bortezomib-MT and 90.7 months in thalidomide-MT (p=0.51).

### 3.3 Impact of front-line treatment on survival

**Figure 3:**
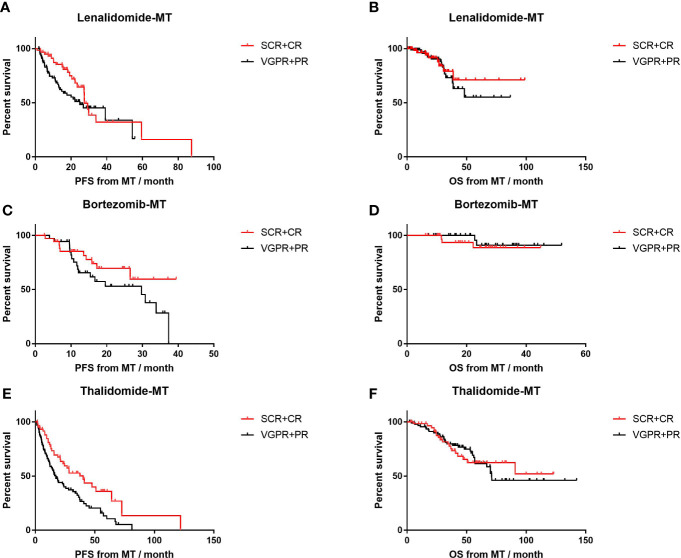
Impact of front-line response depth on progression-free survival (PFS) and overall survival (OS) of lenalidomide, bortezomib, and thalidomide maintenance. **(A, B)** The median PFS and median OS of patients with front-line response of sCR or CR (n=61) versus front-line response of PR or VGPR (n=80) in patients on lenalidomide maintenance. **(C, D)** The median PFS and median OS of patients with front-line response of sCR or CR (n=36) versus front-line response of PR or VGPR (n=43) in patients on bortezomib maintenance. **(E, F)** The median PFS and median OS of patients with front-line response of sCR or CR (n=61) versus front-line response of PR or VGPR (n=94) in patients on thalidomide maintenance.

In patients on thalidomide maintenance, those who achieved CR or sCR before MT had prolonged PFS (median, 40.1 m, n=61) compared to those with VGPR or worse (median, 17.2 m, n=94; p=0.003; HR=0.54, 95% CI 0.37–0.81; **Figure 3**), while PFS was comparable for those with response of CR/sCR and ≤VGPR in lenalidomide-MT (27.4 vs. 25.0 months, p=0.10) and bortezomib-MT (NR vs 29.7m, p=0.16). Patients in each group had similar OS despite different response depths before MT. In the meantime, PFS and OS were not affected by induction regimens in all maintenance groups (**Supplementary Figure S1**).

### 3.4 Impact of baseline cytogenetics on survival

**Figure 4:**
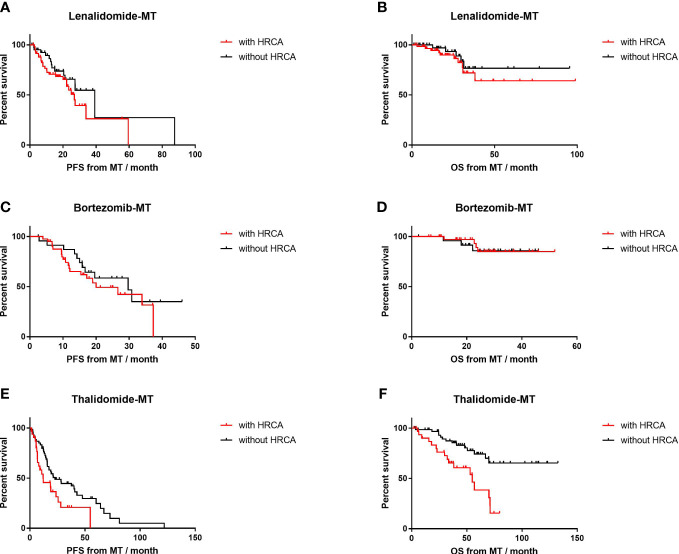
Impact of high-risk cytogenetics on progression-free survival (PFS) and overall survival (OS) of lenalidomide, bortezomib, and thalidomide maintenance. **(A, B)** The median PFS and median OS of patients with high-risk cytogenetics (n=60) versus those without (n=42) in patients on lenalidomide maintenance. **(C, D)** The median PFS and median OS of patients with high-risk cytogenetics (n=41) versus those without (n=25) in patients on bortezomib maintenance. **(E, F)** The median PFS and median OS of patients with high-risk cytogenetics (n=35) versus those without (n=62) in patients on thalidomide maintenance.

For patients with adverse cytogenetic abnormalities (**Figure 4**), thalidomide-MT resulted in significantly impaired PFS (12.1 vs. 22.8 months, p=0.02; HR=1.8, 95% CI 1.0–3.4) and OS (54.9 months vs. NR, p<0.001; HR=3.2, 95% CI 1.5–7.0). In contrast, PFS was comparable in patients with HRCA or not on lenalidomide-MT (26.9 vs. 39.2 months, p=0.19) or bortezomib-MT (20.0 vs. 29.7 months, p=0.36), respectively. OS was not reached in either subgroup. The 4-year survival rate was 83.3% versus 88.1% in the lenalidomide-MT group, and 90.2% versus 88.0% in the bortezomib-MT group, respectively.

To be more specific, 1q21+ on thalidomide-MT was an inferior predictor of shortened median PFS (12.2 vs. 22.8 months, p=0.07) and impaired median OS (54.9 months vs. NR, p<0.01; HR=2.4, 95% CI 1.1–5.5), as shown in **Supplementary Figure S2**. However, in the lenalidomide group, the median PFS was similar for those with 1q21+ or not (26.9 vs. 27.4 months, p=0.84); 4-year OS was 89.4% and 82.5% (p=0.37), respectively. In the bortezomib group with 1q21+ or not, the median PFS was 26.6 vs. 29.7 months (p=0.99); 4-year OS was 96.7% and 84.2% (p=0.14).

In the circumstance of 17p−, thalidomide-MT also resulted in impaired PFS (6.8 vs. 22.8 months, p=0.04; HR=3.0, 95% CI 0.6–16.0) and impaired OS (32.3 vs. 71.1 months, p=0.005; HR=4.0, 95% CI 0.6–26.5). As for lenalidomide-MT, deletion 17p or not did not significantly affect PFS (22.2 vs. 27.4 months, respectively; p=0.11); OS was NR (p=0.73). The median PFS for patients on bortezomib-MT with 17p deletion was shorter, yet comparable to those without 17p− (19.5 vs. 29.7 months, p=0.52), and OS was comparable (NR vs. NR, p=0.12; **Supplementary Figure S3**).

Only one patient presented with high-risk IgH translocation in the thalidomide-MT group, while patients with t(4,14) or t(14,16)) on lenalidomide-MT or bortezomib-MT had similar median PFS and OS as those without (**Supplementary Figure S4**).

In the meantime, PFS and OS were not affected by baseline ISS stage in all maintenance groups.

### 3.5 Impact of maintenance duration on survival

**Figure 5:**
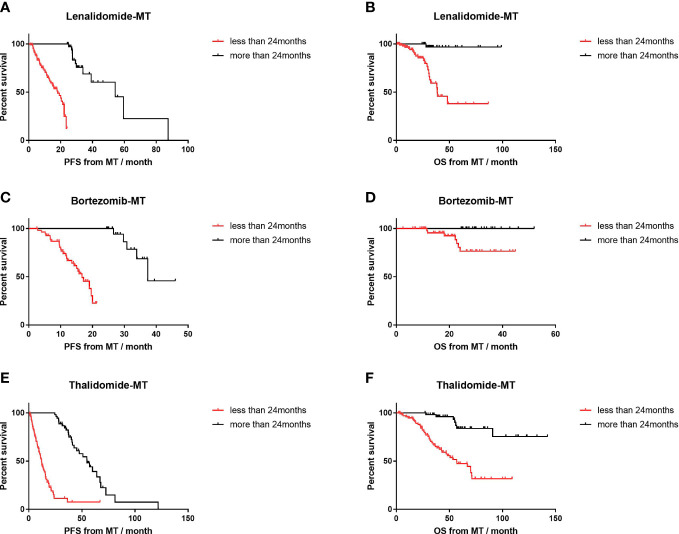
Impact of maintenance duration on progression-free survival (PFS) and overall survival (OS) of lenalidomide, bortezomib, and thalidomide maintenance. **(A, B)** The median PFS and median OS of patients with maintenance duration <24 months (n=103) versus those with longer maintenance (n=38) in patients on lenalidomide maintenance. **(C, D)** The median PFS and median OS of patients with maintenance duration <24 months (n=56) versus those with longer maintenance (n=23) in patients on bortezomib maintenance. **(E, F)** The median PFS and median OS of patients with maintenance duration <24 months (n=101) versus those with longer maintenance (n=54) in patients on thalidomide maintenance.

The duration of maintenance <2 years or longer had a distinct influence on PFS (**Figure 5**). In the lenalidomide-MT group (18.1 vs. 54.3 months, p<0.001; HR=4.9, 95% CI 3.0–8.2), bortezomib-MT group (16.7 vs. 37.3 months, p<0.001; HR=6.3, 95% CI 3.1–12.9), and thalidomide-MT group (12.1 vs. 57.2 months, p=0.001; HR=4.9, 95% CI 3.3–7.4). The results of OS were similar according to maintenance duration.

### 3.6 Multivariate analysis of factors influencing survival

Lenalidomide or bortezomib maintenance had a non-superior impact on survival compared to thalidomide maintenance in the multivariate analysis after adjusting for high-risk cytogenetics by FISH, response depth prior to maintenance, and maintenance duration (**Table 3**). Meanwhile, multivariate analysis by Cox model confirmed that deepened front-line response (CR or sCR) had an independent protective impact on PFS and prolonged maintenance duration on PFS and OS.

**Table 3:** Multivariate analysis by Cox model for lenalidomide or bortezomib vs. thalidomide maintenance.

Variables	Progression-free survival		Overall survival	
	HR (95% CI)	*p*-value	HR (95% CI)	*p-*value
**CR or sCR before MT (vs. <CR)**	0.53 (0.39-0.72)	<0.001	1.20 (0.76-1.90)	0.45
**HRCAs by FISH (vs. standard risk)**	1.30 (0.94-1.81)	0.11	1.27 (0.77-2.11)	0.35
**Maintenance duration <24 months (vs. >24 months)**	11.77 (7.59-18.24)	p<0.001	8.22 (3.93-17.20)	<0.001
**Lenalidomide-MT (vs. thalidomide-MT)**	1.40 (0.89-2.19)	0.15	2.20 (0.85-5.74)	0.11
**Bortezomib-MT (vs. thalidomide-MT)**	1.07 (0.68-1.68)	0.77	2.35 (0.89-6.22)	0.09

## 4 Discussion

Maintenance therapy has been considered as an important part of the whole process management of treatment strategies in multiple myeloma (MM). The depth of response is further improved during maintenance, even switching to MRD negativity. The schema of maintenance for post-transplant patients is relatively perspicuous. However, key questions related to maintenance in non-transplant patients are ambiguous, such as timing to start, approaches, and duration. Oral agents like lenalidomide or ixazomib have been approved for maintenance in transplant-ineligible NDMM patients based on randomized controlled trial (RCT) data, while evidence from real-world study (RWS) is quite inadequate in such population. To address the unmet need of insufficient data, we conducted this multi-centered study to summarize the real-world patient characteristics, maintenance patterns and clinical outcomes in non-transplant NDMM patients in China. Our findings demonstrated that lenalidomide and bortezomib were superior to thalidomide for further improvement of response and achieved benefit of survival regardless of cytogenetic risk.

The special situation in China was that melphalan was not available in China’s mainland until the end of 2018. Even in China’s tertiary hospitals, the ASCT rate was only 15%–30%. Therefore, the majority of patients in our study were younger than 65 years. After being reimbursed in 2017, branded or generic bortezomib and lenalidomide were comprehensively administrated in MM patients. Compared to a further 4.5% improvement of response by thalidomide during maintenance, the boosting of response in bortezomib maintenance was greater, and lenalidomide achieved an extra 10% deepening. In the community-based UPFRONT study, single-agent bortezomib maintenance for only five cycles following bortezomib-based induction therapies improved response depth in approximately 16% of patients (17).The baseline data were of distinct bias in three groups. Only approximately one-third of the patients in thalidomide group presented high-risk characteristics, which was over 50% in the other two approaches. The percentages of any single high-risk cytogenetic abnormality including 1q21+, 17p−, and adverse IGH rearrangements were lower in thalidomide-treated patients so was that of R-ISS 3. Therefore, PFS or OS in lenalidomide or bortezomib groups could be compromised by the selection bias of more high-risk patients. Even so, our data clearly demonstrated that both lenalidomide and bortezomib could reverse the negative impact of high-risk cytogenetics on PFS and OS (**Figure 4**) compared to those with standard-risk. Although the efficacy of lenalidomide as maintenance in high-risk patients was controversial, recent Myeloma XI trial has suggested that high-risk sub-population in lenalidomide maintenance group had significant longer PFS compared to that of placebo (10). While bortezomib is listed as the “other recommended regimen” in most guidelines (18), many trials have proved its advantage in high-risk disease. Mayo Clinic consensus recommended bortezomib for patients with high-risk cytogenetics (6, 11, 19), both in front-line and maintenance settings. By contrast, this study confirmed that thalidomide maintenance could not overcome the inferior impacts of high-risk disease in real-world practice.

An important issue regarding maintenance in non-transplant scenario was patients’ status, mainly front-line regimens and response status. Although maintenance therapy started after 4–12 cycles of induction regimens or if stable disease was obtained in some RCT studies, all patients in our study initiated their maintenance with a median seven cycles of front-line therapy and achieved PR or better. We found that compared to response of PR or VGPR, achieving CR or sCR as before maintenance was an independent protecting factor for PFS (**Tables 3**). Meanwhile, survival outcome in maintenance with novel drugs like lenalidomide or bortezomib was not affected by front-line response (**Figure 3**). In addition, deep response rates during maintenance with lenalidomide or bortezomib were further enhanced yet fell in the thalidomide group. These trends coincided with the results in some RCT studies (7, 20).

The maintenance duration in transplant-ineligible patients is still indefinite. Previous studies demonstrated that lengthy maintenance could result in better survival and depth of response (21, 22). In spite of continuous treatment, such as the FIRST trial, PFS in patients with continuous lenalidomide and dexamethasone (Rd) was 26 months (23), which was similar in our L-MT group (27.4 months). Our data also confirmed that maintenance duration had an independent favorable impact on survival (**Figure 5**). Disease progression was still the main reason for drug withdrawal. Only approximately one-third of patients on thalidomide or bortezomib and one-fourth on lenalidomide could persist to keep medication for more than 2 years. The failure from late progression was mainly due to non-optimal response, which is a problem with compromised induction treatment. Thus, the question in non-transplant patients was not “how long the maintenance will be” but “how long the maintenance could be.” More potent regimens are approved for transplant-ineligible patients such as anti-CD38 antibody, daratumumab, lenalidomide, and dexamethasone (DRd) for continuous administration. Therefore, deeper response even MRD negativity could be achieved and translate into longer PFS.

As a retrospective study, there were limitations to be considered. One was the missing data in cytogenetics, which lost a substantial number of patients for high-risk cytogenetics subgroup analysis. However, only incorporating patients with integrative information also caused bias. We recruited all patients who met the inclusion criteria in this study. A full-time research assistant was responsible for all patients’ follow-up. The missing rate of the whole cohort was <5%. Another limitation was the relatively limited follow-up duration for lenalidomide group and bortezomib group. Lenalidomide and bortezomib were first reimbursed by China’s National Healthcare in September 2017. Therefore, these novel drugs were affordable in most myeloma patients since then.

## 5 Conclusions

In this multi-centered real-world study, lenalidomide, bortezomib, or thalidomide after front-line therapy in non-transplant NDMM patients produced similar PFS and OS. However, patients with lenalidomide or bortezomib comprised a greater proportion of high-risk cytogenetic abnormalities. These HRCAs drag down survival in patients with thalidomide, while lenalidomide and bortezomib remedy the negative effect during maintenance. Clinicians in real practice prefer to recommend lenalidomide or bortezomib as maintenance therapy for patients with HRCAs, while thalidomide is still an option for patients with standard risk. Schema of prolonged maintenance duration improved survival despite maintenance regimens. Furthermore, in clinical settings with limited resources to ASCT, maintenance therapy should be highlighted to delay progression.

## Data availability statement

The original contributions presented in the study are included in the article/**Supplementary material**. Further inquiries can be directed to the corresponding author.

## Ethics statement

The studies involving human participants were reviewed and approved by Institutional Ethics Committee of Peking Union Medical College Hospital, as well as the Institutional Ethics Committee of the participated centers from the Northern China MM Registry. Written informed consent for participation was not required for this study in accordance with the national legislation and the institutional requirements.

## Author contributions

JZ designed the experiment. All authors contributed to clinical data collection. ZZ analyzed the data and prepared the tables and figures. ZZ wrote the first draft and JZ revised the draft. All authors reviewed and approved the revised manuscript.

## Supplementary material

Supplementary Figure 1 Impact of different induction regimens on progression-free survival (PFS) and overall survival (OS) of lenalidomide, bortezomib and thalidomide maintenance. **(A, B)** The median PFS and median OS of patients who received VRD (n=27), B-based (without IMiDs, n=98), L-based (without PIs, n=10) and other regimens (n=6) as induction therapy, on lenalidomide maintenance. **(C, D)** The median PFS and median OS of patients who received VRD (n=8), B-based (without IMiDs, n=69), L-based (without PIs, n=2) and other regimens (n=0) as induction therapy, on bortezomib maintenance. **(E, F)** The median PFS and median OS of patients who received VRD (n=0), B-based (without IMiDs, n=96), L-based (without PIs, n=2) and other regimens (n=57) as induction therapy, on thalidomide maintenance.

Supplementary Figure 2 Impact of 1q21 amplification on progression-free survival (PFS) and overall survival (OS) of lenalidomide, bortezomib and thalidomide maintenance. **(A, B)** The median PFS and median OS of patients with 1q21 amplification (n=47) versus those without (n=57) in patients on lenalidomide maintenance. **(C, D)** The median PFS and median OS of patients with 1q21 amplification (n=30) versus those without (n=38) in patients on bortezomib maintenance. **(E, F)** The median PFS and median OS of patients with 1q21 amplification (n=30) versus those without (n=78) in patients on thalidomide maintenance.

Supplementary Figure 3 Impact of 17p deletion on progression-free survival (PFS) and overall survival (OS) of lenalidomide, bortezomib and thalidomide maintenance. **(A, B)** The median PFS and median OS of patients with 17p deletion (n=16) versus those without (n=78) in patients on lenalidomide maintenance. **(C, D)** The median PFS and median OS of patients with 17p deletion (n=11) versus those without (n=55) in patients on bortezomib maintenance. E, F The median PFS and median OS of patients with 17p deletion (n=6) versus those without (n=102) in patients on thalidomide maintenance.

Supplementary Figure 4 Impact of high-risk IgH translocation on progression-free survival (PFS) and overall survival (OS) of lenalidomide, bortezomib and thalidomide maintenance. **(A, B)** The median PFS and median OS of patients with t(4,14) or t(14,16) (n=15) versus those without (n=76) in patients on lenalidomide maintenance. **(C, D)** The median PFS and median OS of patients with t(4,14) or t(14,16) (n=15) versus those without (n=49) in patients on bortezomib maintenance.
