# Supplementary figures and images for: Lenalidomide or bortezomib as maintenance treatment remedy the inferior impact of high-risk cytogenetic abnormalities in non-transplant patients with newly diagnosed multiple myeloma: a real-world multi-centered study in China
Source: Front Oncol. 2023 Apr 20;13:1028571. doi: 10.3389/fonc.2023.1028571 (PMC10157094; doi:10.3389/fonc.2023.1028571)

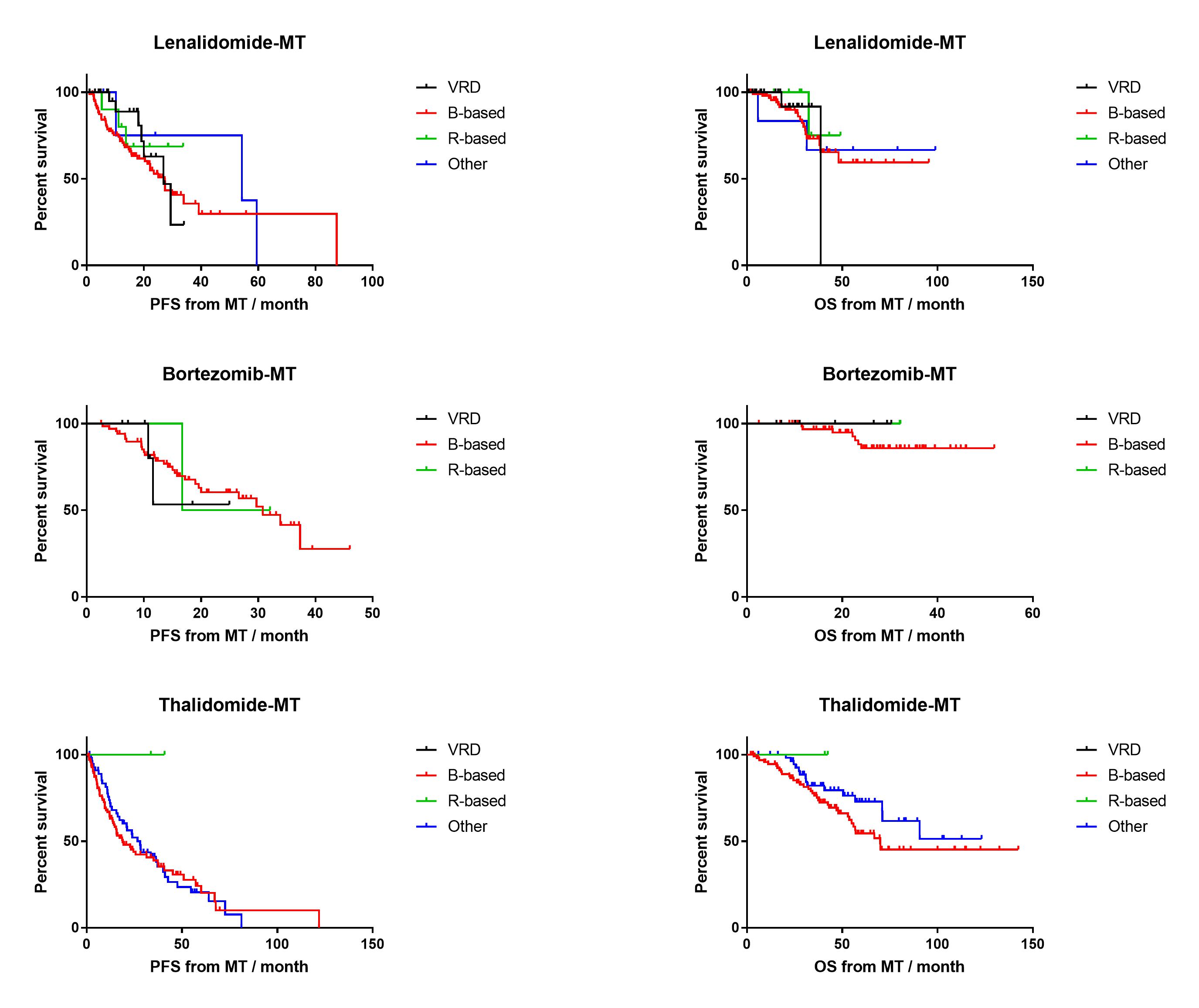

Supplement: Supplementary Figure 1 — Impact of different induction regimens on progression-free survival (PFS) and overall survival (OS) of lenalidomide, bortezomib and thalidomide maintenance. (A, B) The median PFS and median OS of patients who received VRD (n=27), B-based (without IMiDs, n=98), L-based (without PIs, n=10) and other regimens (n=6) as induction therapy, on lenalidomide maintenance. (C, D) The median PFS and median OS of patients who received VRD (n=8), B-based (without IMiDs, n=69), L-based (without PIs, n=2) and other regimens (n=0) as induction therapy, on bortezomib maintenance. (E, F) The median PFS and median OS of patients who received VRD (n=0), B-based (without IMiDs, n=96), L-based (without PIs, n=2) and other regimens (n=57) as induction therapy, on thalidomide maintenance. [file Image_1.jpeg]

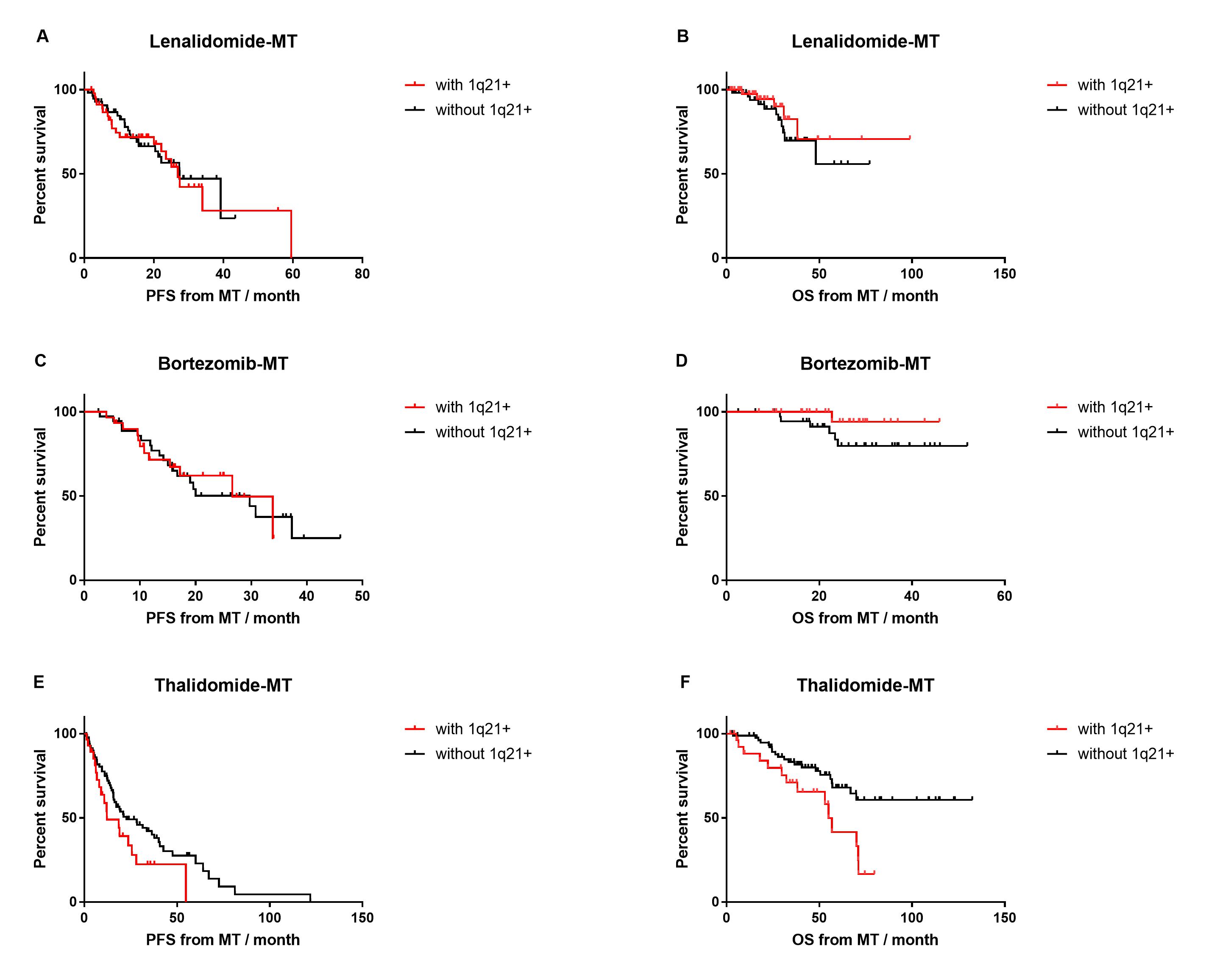

Supplement: Supplementary Figure 2 — Impact of 1q21 amplification on progression-free survival (PFS) and overall survival (OS) of lenalidomide, bortezomib and thalidomide maintenance. (A, B) The median PFS and median OS of patients with 1q21 amplification (n=47) versus those without (n=57) in patients on lenalidomide maintenance. (C, D) The median PFS and median OS of patients with 1q21 amplification (n=30) versus those without (n=38) in patients on bortezomib maintenance. (E, F) The median PFS and median OS of patients with 1q21 amplification (n=30) versus those without (n=78) in patients on thalidomide maintenance. [file Image_2.jpeg]

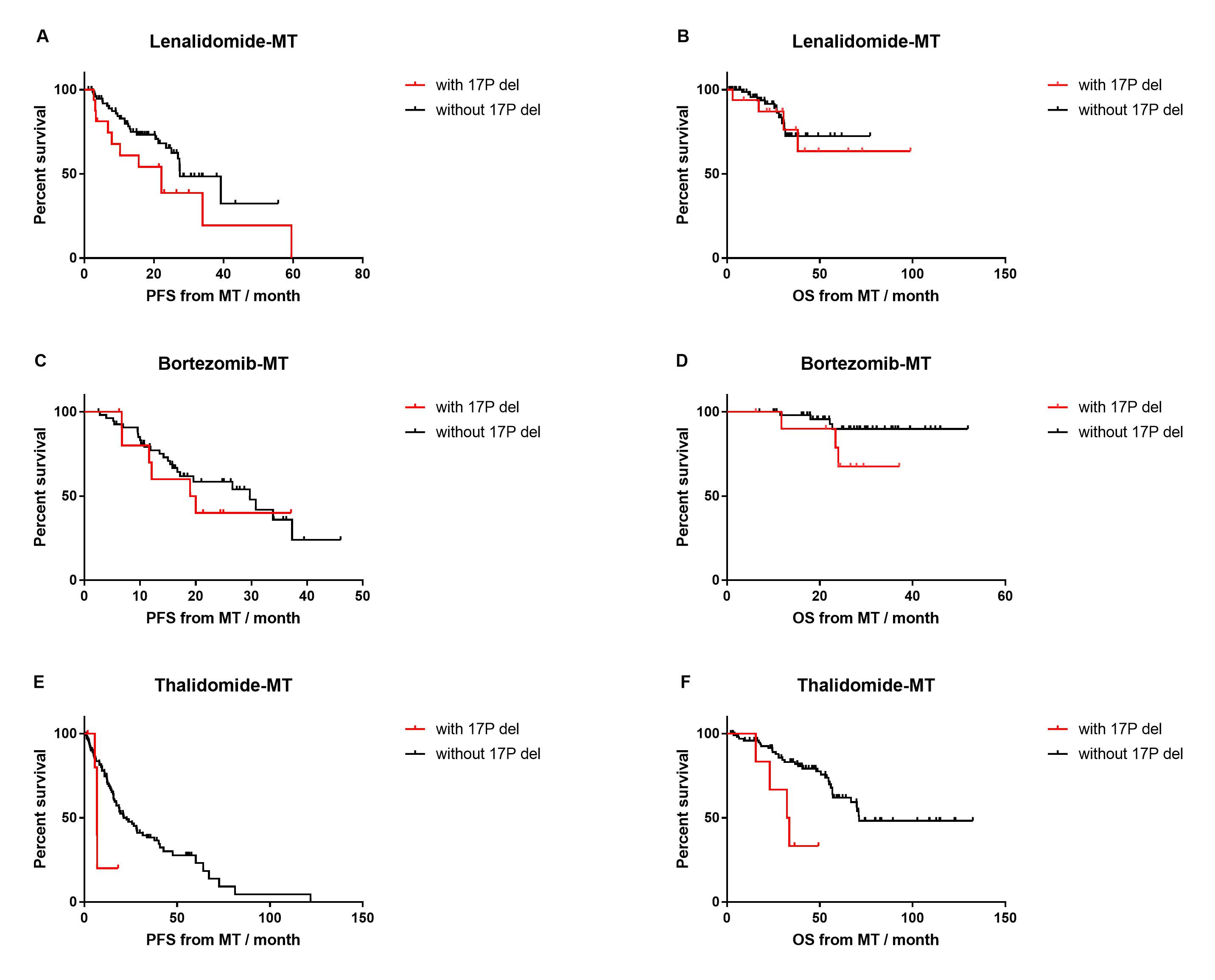

Supplement: Supplementary Figure 3 — Impact of 17p deletion on progression-free survival (PFS) and overall survival (OS) of lenalidomide, bortezomib and thalidomide maintenance. (A, B) The median PFS and median OS of patients with 17p deletion (n=16) versus those without (n=78) in patients on lenalidomide maintenance. (C, D) The median PFS and median OS of patients with 17p deletion (n=11) versus those without (n=55) in patients on bortezomib maintenance. E, F The median PFS and median OS of patients with 17p deletion (n=6) versus those without (n=102) in patients on thalidomide maintenance. [file Image_3.jpeg]

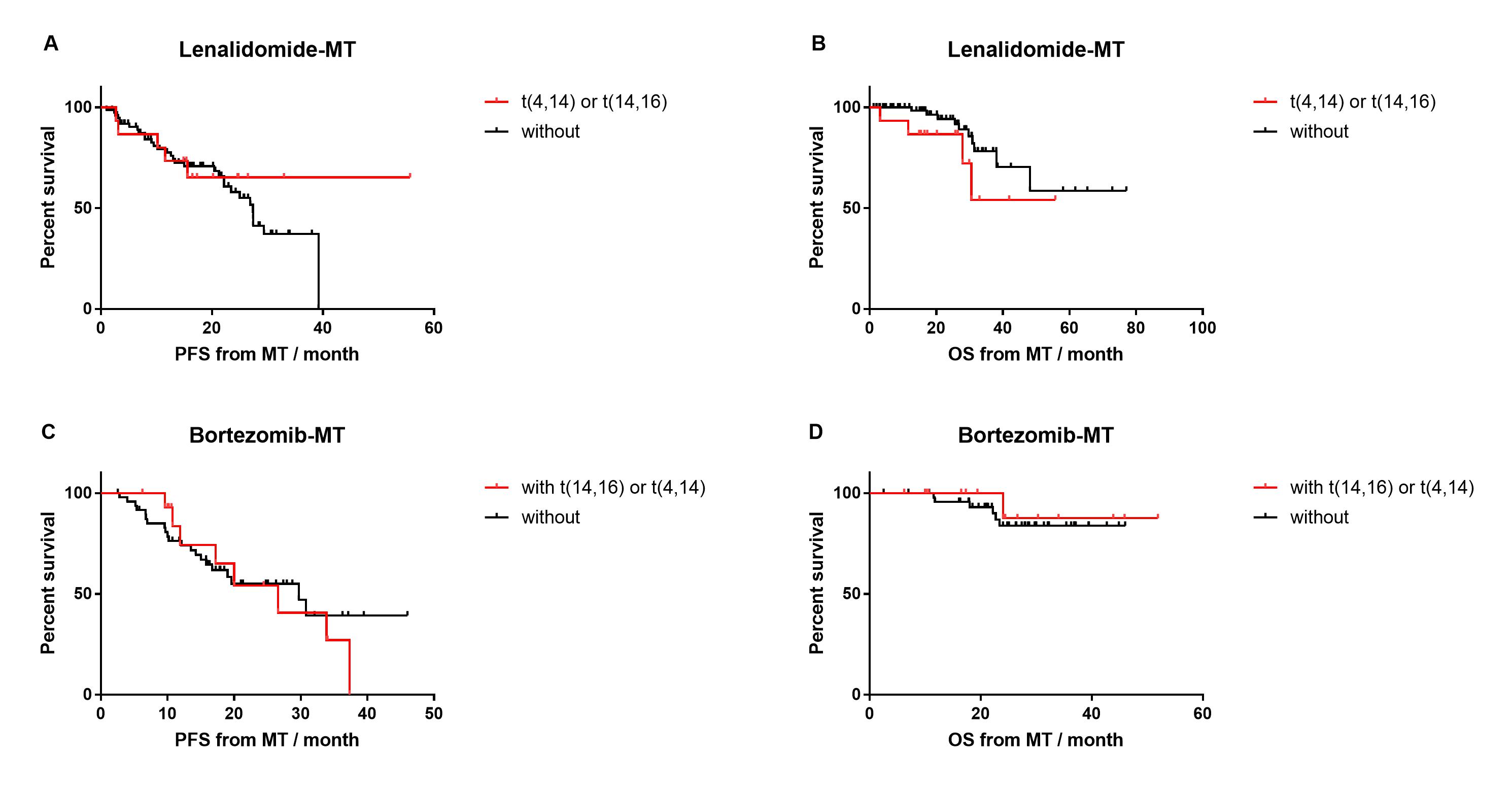

Supplement: Supplementary Figure 4 — Impact of high-risk IgH translocation on progression-free survival (PFS) and overall survival (OS) of lenalidomide, bortezomib and thalidomide maintenance. (A, B) The median PFS and median OS of patients with t(4,14) or t(14,16) (n=15) versus those without (n=76) in patients on lenalidomide maintenance. (C, D) The median PFS and median OS of patients with t(4,14) or t(14,16) (n=15) versus those without (n=49) in patients on bortezomib maintenance. [file Image_4.jpeg]
